# Sunflower Citizens? Resilience as Lived Negotiation among Mental Health Professionals in Zero-COVID China

**DOI:** 10.1007/s11013-026-10007-2

**Published:** 2026-07-09

**Authors:** Anna Iskra

**Affiliations:** Department of Applied Social Sciences, Hong Kong Polytechnic University, Hong Kong, China

**Keywords:** Resilience, Adaptability, Zero-COVID, Psychological counselling, Therapeutic governance

## Abstract

This article examines how resilience and its key tool, adaptability, were defined, mobilized, and contested during Shanghai’s Zero-COVID period, foregrounding the understudied perspectives of psychological counsellors. Drawing on 30 interviews with 25 counsellors and a discursive analysis of 268 posts from the Shanghai Mental Health Centre (SMHC), it shows how state discourse framed resilience as optimism, acceptance, and disciplined self-adjustment, while counsellors reinterpreted it through trauma, helplessness, delayed collapse, and relational care. As intermediaries increasingly woven into state institutions through outsourcing arrangements, counsellors negotiated between official prescriptions and the lived crises of clients whose distress was shaped by uncertainty, family conflict, gendered burdens, and economic precarity. By tracing these quiet negotiations, the article argues that resilience in Shanghai was neither a universal trait nor simply a neoliberal injunction. Rather, it functioned as a culturally situated, politically charged field of interpretation through which mental health, citizenship, and crisis survival were negotiated. Situating Shanghai within wider debates on resilience, the article shows how resilience became a “multiple”: at once demanded, embodied, contested, and re-signified through the interactions of state institutions, professional ethics, and the uneven temporalities of crisis.

## Introduction

“Wishing you, like the sunflower, always to seek the sunlight”(from the article on psychological resilience during the COVID-19 pandemic, posted on the official WeChat account of the Shanghai Mental Health Centre)

“I don’t know whether to call it ‘adaptation’ or ‘helpless forced acceptance’…” (psychological counsellor, woman, 43 Int 20)

During China’s Zero-COVID period, psychological resilience and adaptability became ubiquitous idioms through which mental health, crisis survival, and citizenship were publicly discussed. State-affiliated mental health institutions, media accounts, and educational campaigns promoted emotional stability, optimism, and flexibility as appropriate responses to prolonged uncertainty. Yet, as the counsellor quoted above suggests, what official discourse framed as resilience could also be experienced as exhaustion, resignation, or reluctant endurance. The contrast between a sunflower turning toward the sun and a practitioner’s uncertainty about whether she had adapted or merely submitted captures the contested moral and political life of these terms.

This article examines how resilience and adaptability were interpreted and negotiated among psychological counsellors in Shanghai during the COVID-19 pandemic. Rather than treating resilience as a universal psychological trait, I use it as an analytic entry point into a cluster of locally circulating categories, including *xinli renxing* (psychological toughness/resilience), *kangnili* (resistance force), *xinli tanxing* (psychological elasticity), and *shiyingxing* (adaptability). These terms partially overlap with English-language discussions of resilience, but they do not map neatly onto a single underlying psychological “thing.” Their meanings were actively shaped through interactions among state institutions, mental health professionals, and clients trying to make sense of life in Zero-COVID Shanghai.

The article focuses on psychological counsellors as mediating experts positioned between official mental health discourse and the everyday experiences of distress among urban residents. Counsellors occupy an ambivalent location within China’s rapidly expanding psychological-services sector: many operate through privatized and digitally mediated markets yet increasingly participate in state-sponsored initiatives through outsourcing and community crisis response. During the pandemic, this intermediary role became especially pronounced. Counsellors encountered both the official promotion of psychological toughness and flexibility as civic virtues and the emotional realities of clients navigating confinement, uncertainty, family strain, and social disruption.

Examining these dynamics shows that resilience under Zero-COVID functioned neither as a purely top-down instrument of governance nor as a straightforward language of individual coping. Instead, it emerged as a contested interpretive vocabulary through which mental health professionals negotiated the relationship between psychological well-being, social responsibility, and political authority. Counsellors often adopted the language of resilience and adaptability while subtly reframing it through clinical idioms of trauma, numbness, delayed breakdown, gendered exhaustion, and relational care. Official discourse was thus neither simply reproduced nor openly rejected but reworked in practice (Iskra, 2023), as counsellors selectively appropriated official psychological vocabularies while inflecting them with their own ethical and therapeutic priorities.

Over the past two decades, resilience has become one of the most widely invoked and hotly contested concepts across sociology, political science, and anthropology. Its popularity lies in its semantic flexibility: it can mean bouncing back, adapting to adversity, or transforming in response to crisis. These debates have generated important insights, but three gaps remain especially striking.

First, most scholarship on resilience as governance has focused on Euro-American settings, leaving relatively little ethnographic analysis of how resilience discourse operates in political environments shaped by different institutional histories.

Second, while existing studies have examined resilience either as a policy discourse or as a lived experience, they have paid comparatively little attention to the “middle level”: counsellors and other practitioners who mediate between policy language, professional ethics, and clients’ suffering. Insights from studies of frontline professionals are particularly useful here. Lipsky’s (2010) concept of “street-level bureaucracy” highlights how practitioners inevitably interpret and modify official policies in everyday practice, a dynamic reflected in how Shanghai counsellors translated state discourse on resilience into therapeutic strategies shaped by clinical ethics and clients’ lived realities. Brodwin’s (2013) work on community psychiatry similarly shows that mental health professionals operate within institutional mandates while negotiating moral obligations to those they serve, illuminating how counsellors in this study mediated between state expectations of emotional regulation and the suffering they encountered in practice. These actors are not passive conduits; they actively reinterpret, contest, and reshape the meaning of resilience in their professional and personal lives.

Third, the embodied, relational, and gendered dimensions of resilience remain undertheorized in broader sociological accounts. While anthropologists have emphasized these aspects in localized case studies (Eitel, 2023; Kenney & Müller, 2024; Zraly & Nyirazinyoye, 2010), they are rarely connected to wider debates on resilience as governance. As a result, we still know relatively little about how resilience operates simultaneously as a political project and a lived, unevenly distributed practice.

This article addresses these three gaps by examining psychological counsellors in Shanghai during the Zero-COVID period as affective intermediaries situated between state mental-health agendas and the emotional realities of everyday life. Drawing on interviews with practitioners alongside discourse analysis of mental health materials produced by the Shanghai Mental Health Centre (SMHC), it traces how psychological toughness, flexibility, and adaptability circulated through overlapping institutional and professional arenas. Rather than asking how “Chinese resilience” differs from “Euro-American resilience,” the article asks how mental health categories became politically charged idioms through which counsellors, clients, and institutions negotiated the relationship between psychic survival and policy compliance.

The study makes three contributions. First, it shows that resilience in Shanghai cannot be understood simply as an imported neoliberal ideology or as a uniquely Chinese form of collective endurance. Instead, it operated as a flexible interpretive framework whose meanings were continually renegotiated within specific institutional and moral worlds. Second, it highlights counsellors as mediating experts who simultaneously reproduced, reinterpreted, and sometimes unsettled official psychological narratives. Third, it foregrounds the embodied, relational, and gendered dimensions of resilience as lived experience, showing how adaptation during the pandemic often involved emotional labor, bodily strain, mutual aid, and delayed psychological collapse.

In this sense, resilience in Shanghai appears less as a stable trait than as what Zebrowski (2025) calls a “resilience multiple”: a concept invoked for divergent purposes across political, institutional, and everyday contexts. Under Zero-COVID, resilience was at once promoted by state-affiliated institutions, mobilized in therapeutic practice, and quietly questioned by those asked to embody it.

## Resilience as Governance and Lived Practice

Across the social sciences, resilience has increasingly been interpreted not simply as a psychological capacity but as a political technology for managing uncertainty and crisis. Critical scholars argue that resilience emerged alongside transformations in governance that shift responsibility for managing risk from institutions to individuals and communities. In this perspective, resilience encourages adaptation to instability rather than addressing its structural causes and can normalize conditions of crisis by framing flexibility and self-adjustment as civic virtues (Chandler, 2014; Joseph, 2013; Reid, 2012). These critiques understand resilience as a form of governmentality (Foucault, 1988), encouraging subjects to regulate themselves under conditions of uncertainty. Adaptability often functions as the practical expression of this logic, relocating responsibility for coping with crisis onto individuals rather than institutions.

At the same time, resilience is not a singular or ideologically fixed concept. Scholars have shown that it is mobilized across diverse political and institutional projects and may refer to individual coping, institutional adaptation, or broader forms of social transformation (Bourbeau, 2018; Zebrowski, 2025). Rather than treating resilience as inherently emancipatory or inherently neoliberal, these perspectives emphasize its multiplicity and the importance of examining how it is defined and deployed in particular contexts.

In anthropological scholarship, Andaya and Cooper (2025) identified two dominant approaches to resilience. First, anthropologists have examined resilience as a lived social process, focusing on how individuals, communities, and ecosystems endure hardship and pursue well-being amid crises. This body of work explores resilience through culturally specific practices, values, relationships, and material conditions. Examples include studies of caregiving strategies for people living with AIDS (Manderson et al., 2016), Rwandan women recovering from genocidal violence (Zraly & Nyirazinyoye, 2010), or the adaptation to climate change among the rural inhabitants of the Arctic (Crate, 2013). Collectively, this scholarship emphasizes that resilience is not a universal psychological trait, but a phenomenon shaped by social, political, cultural, and ecological contexts. It builds on earlier anthropological concerns with concepts such as adaptation (Rappaport, 1968), resistance (Ortner, 1995), survivance (Hassan, 2023), and collective continuance (Clark et al., 2022), highlighting how people creatively sustain meaningful lives under conditions of uncertainty and adversity.

The second approach treats resilience as a policy discourse and technology of governance. Anthropologists working in this tradition investigate how governments, institutions, and international organizations deploy resilience as a policy framework, particularly in contexts such as climate adaptation (Hastrup, 2009), displacement and migration (Oliver-Smith, 2016), and military planning (Jauregui, 2015). Ethnographic research demonstrates how resilience discourse can shift responsibility for managing risk and insecurity from states to individuals and local communities (Barrios, 2016). Scholars have also criticized the common metaphor of “bouncing back” for normalizing precarity and inequality by presenting existing conditions as desirable states to which societies should return (Evans & Reid, 2014). This work aligns with broader critiques that view resilience as a vehicle for neoliberal governance and argue that such interpretations risk overlooking the concept’s social significance and the diverse meanings it holds for different communities (Bollig, 2014).

These perspectives foreground three crucial insights: First, resilience is affective and embodied, experienced through emotions, bodily strain, and temporal rhythms. Second, it is relational, emerging in networks of care, mutual aid, and gendered labor. Third, it is culturally variable, shaped by histories, moral frameworks, and political economies.

Taken together, these debates suggest that resilience is not monolithic but contested. It can function as a discourse that reframes structural suffering as a matter of self-management, yet it can also be reappropriated as critique, reform, or ordinary survival. At the same time, much scholarship treats resilience either as a macro-level governance strategy or as a diffuse background condition of everyday life. Less attention has been paid to resilience as a contested category of practice actively negotiated by policymakers, practitioners, and citizens. Mental health professionals are especially important here. Positioned between institutional expectations and clients’ emotional realities, they mediate the translation of psychological concepts into lived worlds.

In the Chinese context, these dynamics unfold within a rapidly expanding psychological-services sector shaped by postsocialist reforms, marketization, and state interest in emotional governance. Scholars have examined how psy knowledge circulates through urban life, self-cultivation, and family relations (Huang, 2014; Yang, 2015, 2017; Zhang, 2015, 2017, 2020). Yet, relatively little research has explored how counsellors themselves negotiate the political implications of their work, particularly during crisis (ex. Iskra, 2026). Recent scholarship on popular responses to Zero-COVID further shows that compliance, accommodation, and critique were far more complicated than a simple opposition between obedience and resistance (Cai & Mason, 2022; Ma et al., 2025). This article extends those insights into the domain of mental health by examining how counsellors interpreted psychological toughness, flexibility, and adaptability amid lockdowns.

Rather than assuming that resilience operated uniformly across contexts, the analysis below shows how its meanings were continually negotiated within specific institutional, cultural, and political environments. It contributes to anthropological discussions of resilience by shifting attention from resilience as either governance or lived experience to the actors who mediate between these domains. It shows how counsellors function as affective intermediaries who reproduce, reinterpret, and at times challenge official expectations of adaptation and psychological well-being. Beyond anthropology, it demonstrates that resilience is not simply implemented as policy or experienced as a personal capacity, but actively translated through professional practice. Examining these processes during Zero-COVID reveals resilience as a politically and morally contested concept whose meanings remain unstable and context-dependent.

## Research Demographic and Methods

Between September 2022 and May 2023, I conducted online and in-person fieldwork with 25 counsellors in Shanghai’s psychological-services sector. Because travel restrictions limited access to mainland China during much of the research period, participants were initially recruited through 525 Psychology Net, a platform connecting counsellors with clients, and later through snowball sampling. Interviews were conducted online and, following the reopening of borders in spring 2023, in person.

In total, this multi-stage process generated 30 semi-structured interviews, ranging from exploratory discussions of everyday practice to in-depth reflections on professional and personal challenges during the pandemic. The interlocutors—19 women and 6 men, aged 22–66—reflected the gendered composition of the counselling milieu in China. Only seven participants held university degrees in psychology; most entered the profession through short-term certificate programs that proliferated after the early 2000s. Therapeutic repertoires varied, with psychodynamics, cognitive-behavioral, and family therapy most cited. What united them was their positioning in the loosely regulated, digitally mediated, and market-driven “psycho-boom” (Huang, 2014) that has reshaped China’s mental health landscape over the past two decades. All participants are anonymized; Table 1 outlines demographics, training, professional role, therapeutic orientation, and clientele.

**Table 1.** List of interviewees

No.	Interview date	Gender	Age	Birthplace	Tertiary level education in psychology /counselling	Occupation and workplace	Professional experience in mental health	Therapeutic approach	Client base
1.	18.09.2022	F	21	Zunyi, Guizhou province	yes (BA)	Product development and psychological consultancy for a mental health app	1 year	Cognitive behavioral therapy (CBT)	Adults
2.	21.09.2022	F	28	Small town in Sichuan province	yes (MA)	Part-time counsellor for a counselling center, full-time counsellor for a mental health app	1.5 years	CBT, art therapy, psychoanalysis	Children
3.	24.09.2022	M	66	Shanghai	unknown	Individual counselling practice	10 years	Cognitive therapy	Adults
4.	a.25.09.2022 b. 23.05.2023	F	44	Zhejiang province	yes	Part-time counsellor for a counselling center, full-time job in finance	8 years	Talk therapy (unspecified approach)	Children, teenagers, adults
5.	26.09.2022	F	55	Shanghai	no	Counsellor working for two counselling centers	4 years	CBT	Young adults
6.	27.09.2022	M	43	Xinjiang Uygur Autonomous Region	yes	Individual counselling practice	20 years	Psychoanalysis, psychodynamics, analytical psychology, cognitive therapy	Adults
7.	28.09.2022	F	35	Rural Henan province	yes (enrolled in an MA program)	Individual counselling practice	6 years	Humanistic psychology, positive psychology, psychoanalysis, body-centered psychotherapy, Jungian psychology	Adults
8.	29.09.2022	F	37	Shanghai	yes (MA)	Individual counselling practice	12 years	Psychodynamics, Chinese traditional culture and philosophy	Adults
9.	30.09.2022	M	39	County seat town in Heilongjiang province	yes (MA)	Product designer for a counselling center, part-time counsellor for a different counselling center	4-5 years	Psychodynamics, family therapy	Adults
10.	2.10.2022	F	38	Hubei province	no	Product manager of a counselling and mental health education center that serves private companies and government organizations such as the Woman’s Federation or Party committees (*dang wei*)	N/A	N/A	Adults
11.	3.10.2022	F	44	Lanzhou, Gansu province	no	Individual counselling practice	8 years	Psychoanalysis, CBT, gestalt, hypnosis, OH cards	Children, teenagers, adults
12.	4.10.2022	F	mid-30s	Town in Jiangsu province	yes (MA)	Counsellor for a counselling center serving private companies and government organizations	2 years	Psychodynamics	adults
13.	7.10.2023	F	32	Hangzhou, Zhejiang province	yes (MA)	Individual counselling practice	8 years	Art therapy	Children, teenagers, adults
14.	8.10.2022	F	69	Shanghai	no	Counsellor for a government organization for protection of women and children	16 years	Talk therapy (unspecified)	adults
15.	10.10.2022	F	40	Shanghai	yes (BA)	Individual counselling practice	6 years	Psychoanalysis, humanistic psychology	Children, adults
16.	12.10.2022	F	38	Shanghai	no	Individual counselling practice	3 years	Psychoanalysis, sandplay therapy, OH cards	adults
17.	14.10.2022	F	46	Mid-size city in Anhui province	no	Individual counselling practice, part-time counselling for a counselling center, part-time social work	8 years	Psychodynamics, family therapy	Children, teenagers, adults
18.	a. 01.11.2022 b. 26.05.2023	F	29	Changchun, Jilin province	yes (MA)	Individual counselling practice, including oversees clients, part-time counselor at a university	2 years	CBT, acceptance and commitment therapy (ACT), dialectical behavioral therapy (DBT)	Adults
19.	7.11.2022	F	30	Hua county, Henan province	yes (BA and MA)	Psychological counsellor for a national e-sports team	3 years	Sport psychology	Teenagers, adults
20.	4.11.2022	F	43	Wenzhou, Zhejiang province	no	Counsellor for two counselling centers	5 years	Psychodynamics, sandplay therapy, art therapy	Teenagers, adults
21.	a. 07.11.2022 b. 27.05.2023	M	42	Shanghai	no	Individual counselling practice, occasional referrals from a state hospital	9 years	Family therapy, humanistic psychology, CBT	Adults
22.	30.11.2022	F	39	Harbin, Heilongjiang province	yes (BA, MA), enrolled in a PhD program	Part-time sport counsellor, PhD candidate in sport psychology	17 years	Sport psychology	Teenagers, adults
23.	09.12.2022	M	41	Shanghai	no	Counsellor for the Public Complaints and Proposals Administration Office, private practitioner	10 years	Talk therapy (unspecified)	Adults
24.	a. 14.12.2022 b. 25.05.2023	M	41	Jiangsu province	yes (BA)	Individual counselling practice	9 years	Morita therapy, specialization in OCD treatment	Children, teenagers, adults
25.	18.12.2022	F	43	Village near Shanghai	no	Part-time psychological counsellor	5 years	Talk therapy (unspecified)	adults

The two phases of interviewing captured different moments in the pandemic’s aftermath. Interviews conducted in late 2022 more often emphasized exhaustion, uncertainty, and disrupted practice, whereas follow-up interviews in spring 2023 foregrounded lingering psychological effects, retrospective critique, and economic fallout. This temporal contrast suggests that resilience was experienced not as a stable capacity but as a shifting interpretive framework through which counsellors made sense of both crisis and recovery.

The contemporary position of psychological counsellors in China reflects the rapid expansion and uneven professionalization of the psychological-services sector since the early 2000s. Following SARS, national mental-health initiatives promoted psychological expertise, while the introduction of a national certification system enabled large numbers of non-psychology majors to enter the profession through short-term training programs. The resulting psycho-boom produced a large but highly heterogeneous workforce operating across private academies, digital platforms, and psychiatric institutions. The abolition of the national certification system in 2018 further contributed to fragmented professional pathways and uneven standards (Huang, 2022).

Questions of *bentuhua* (localization) have become central to professional debates, as counsellors negotiate between imported therapeutic models and local moral, cultural, and institutional expectations (Zhang, 2014). These discussions are also shaped by the state’s growing reliance on psychological services for community outreach, crisis intervention, and social management. Counsellors therefore occupy an intermediary position: many are trained through market-driven and digitally mediated pathways yet increasingly participate in state-linked projects of psychosocial governance. Their experiences during Zero-COVID offer a valuable perspective on how official discourses of psychological toughness, flexibility, and adaptability were translated, negotiated, and sometimes contested in practice.

Localization is also entwined with the state’s growing reliance on psychological services for governance. Government departments, trade unions, and community organizations increasingly outsource psychological work, often expecting crisis-management or “stability-maintenance” (*weiwen*) functions rather than individualized psychotherapy. In this context, localization becomes both a practical accommodation to family-centered moral expectations and an alignment with state visions of social harmony and emotional regulation.

This history has produced a dispersed workforce increasingly drawn into state-linked initiatives, especially in community outreach and crisis response. Counsellors thus occupy an intermediary position: trained through market-driven and digitally mediated pathways, yet increasingly enlisted into projects of psychosocial governance. Their perspectives illuminate how grassroots interpretations of the pandemic’s mental-health effects both resonated with and complicated official narratives, and how psychological toughness, flexibility, and adaptability were reworked in therapeutic encounters and in negotiations of precarity and professional identity.

Alongside ethnography, I conducted a discursive analysis of 268 pandemic-related mental health articles published between January 2020 and December 2023 on two official SMHC WeChat accounts: Shanghai Mental Health Center and SMHC Flying Green Ribbon (FGR). SMHC, one of China’s foremost psychiatric institutions, not only treats mental disorders but provides counselling, runs a public hotline, and coordinates post-disaster interventions. Its WeChat communications serve as an important channel for state-sanctioned discourses on mental health and resilience during the pandemic. Several interviewees had trained or collaborated with the Center.

The Flying Green Ribbon platform, launched in April 2014, functions as SMHC’s public education channel, offering preventive guidance, information on mental disorders, patient rights, and relevant policies. It has played a key role in public health outreach and crisis response, coordinating medication assistance for patients with mental illnesses and distributing self-help and psychosocial resources during lockdowns. Content is delivered through diverse formats, including comics, short films, animated or live-streamed lectures, and Q&A sessions with specialists.

**“Please Limit Your Soul’s Desire for Freedom.” The Shanghai Lockdown and Local Mental Health Infrastructures**

The Shanghai lockdown is examined here as a paradigmatic case of how resilience, adaptation, and mental-health infrastructures were mobilized and experienced under Zero-COVID. Beyond its scale, it exposes the contradictions of state-promoted resilience: framed as optimism, discipline, and self-regulation, yet lived as coercion, exhaustion, and forced compliance. Tracking the institutional, commercial, and grassroots mental health landscape shows how psychological support became both a coping resource and a tool of governance, revealing tensions within official resilience discourse.

Unlike Wuhan in early 2020, Shanghai’s two-month lockdown in spring 2022 unfolded with prior experience and stronger infrastructure. Yet the shutdown of a city of 25 million still produced widespread suffering. A district-level closure abruptly expanded on April 1 into a citywide lockdown; unprepared households scrambled for supplies as grocery platforms collapsed and neighborhood offices (*jiedaoban*) failed to cope (Ling, 2023). The pressures of confinement quickly exposed the limits of state resilience rhetoric. Officials forced entry into homes; children were separated from parents; pets were killed during “decontamination” (Zhang, 2023). Elderly and chronically ill residents died after being denied timely medical care, including a nurse who suffered a fatal asthma attack on March 29 when her own hospital refused her entry (Ling & Zhang, 2023). Such scenes starkly contradicted the slogan “People first, lives first.”

The emblematic phrase “control your soul’s desire for freedom” (*kongzhi linghun dui ziyou de kewang*), coined by Xie Bin of the Shanghai Mental Health Center (SMHC), crystallized the lockdown’s psychic stakes. A drone broadcasting it over apartment complexes captured the intrusion of authority into domestic space (L.G., 2022). The moment also exposed SMHC’s ambivalent status: Shanghai’s leading psychiatric authority, yet still colloquially marked by its address—600 Wanping South Road—as a symbol of mental illness. Counsellors noted ongoing efforts to counter this stigma, including SMHC’s recent consumer items such as “600” mooncakes. This dual status shaped reception of its pandemic messaging: calls for emotional restraint invoked expertise but also the shame that deters help-seeking.

It was against this backdrop that mental-health infrastructures—official, commercial, and grassroots—were mobilized with unprecedented intensity, forming an ecology I reconstruct here through interviews with counsellors. The Shanghai Mental Health Center expanded its public communication efforts and widely promoted its 24-hour psychological support hotline: 962525. Beyond SMHC, a dense assemblage of psychological aid services proliferated. Government agencies, universities, hospitals, NGOs, commercial platforms, and private practitioners launched hotlines, online support groups, livestreamed lectures, and self-help materials. Counsellors described colleagues producing short videos with techniques for self-soothing (“give yourself a hug”), recording guided breathing exercises, or posting psycho-educational infographics through WeChat public mental health accounts. Yet the surge in services also exposed infrastructural limits. Several counsellors recounted overwhelming demand on hotlines, inconsistent triaging, and widespread confusion among the public about the purpose of psychological aid, exacerbated by an official’s suggestion that hotline staff could “soothe you to sleep.”

The lockdown also reshaped the counselling industry. Practitioners abruptly shifted online, often losing clients who lacked privacy at home, preferred face-to-face therapy, or deprioritized counselling amid economic precarity. These losses were only partially reversed after reopening. Some centers downsized or closed; others survived only by cutting fees. For counsellors working with children, therapy interruptions were particularly acute. At the same time, several interlocutors noted that the pandemic increased public awareness of mental health, especially among youth, contributing to lasting changes in help-seeking patterns.

Community-level and civil-society initiatives formed another layer of this ecology. Counsellors working with community organizations, the Communist Party-affiliated Woman’s Federation, and neighborhood committees described providing phone-based crisis intervention, including suicide-risk assessment and, in severe cases, activating the vertically integrated administrative chain to enable rapid in-person response even during lockdown. In makeshift hospitals, counsellors offered free-of-charge remote psychological support to quarantined patients and fatigued volunteers. Civil-society actors, mutual-aid groups, and private platforms organized psycho-educational lectures on emotional regulation, family conflict, anxiety prevention, and post-lockdown communication skills.

Despite this expansion, significant ambivalence remained. Counsellors frequently described the field as “chaotic,” marked by uneven training, blurred boundaries between psychological and practical support (e.g., helping families find milk powder or diapers), and the impossibility of addressing emotional distress when basic needs, food, medication, shelter, were unmet. One counsellor characterized psychological services during the lockdown as “painkillers,” capable of containing acute distress but rarely equipped to confront the structural conditions producing it.

This wider context is important because it situates psychological discourse within a broader ecology of compliance, improvisation, and strain. As studies of Zero-COVID have shown, public responses were shaped not only by coercion but also by moral expectations, infrastructural dependence, and what Cai and Mason (2022) call “the big environment,” within which ordinary people assessed both risk and obligation. By late May, as authorities eased restrictions and replaced “lockdown” (*fengcheng*) with euphemisms like “static management” (*jingtai guanli*), frustration crystallized in the blank placard protests, where holding an empty sheet of paper became a powerful indictment of censorship and psychic containment (Xu, 2024). The Shanghai lockdown thus reveals how resilience and mental health became entangled in crisis governance: not simply imposed from above, but negotiated, improvised, and contested across institutional, professional, and everyday domains.

## Sunflower Citizens: Resilience and Adaptability in Shanghai Mental Health Centre’s Public Messaging

The collection of pandemic-era communiqués from SMHC’s official WeChat accounts (2020–2023) offers a window into how state-affiliated mental health messaging framed psychological response under conditions of extraordinary social control. These materials did not simply promote “resilience” in an abstract sense. Rather, they circulated a vocabulary of psychological toughness, elasticity, acceptance, gratitude, and disciplined adjustment that linked mental health to appropriate conduct during crisis. Instead of treating these categories as exact equivalents of English-language resilience discourse, I analyze them as locally meaningful terms whose circulation reveals how emotional life was rendered governable under Zero-COVID.

### Conceptualizing Resilience and Adaptability: The Chinese Lexicon of Endurance

The SMHC materials employ a rich vocabulary to describe the ideal psychological posture for navigating the Zero-COVID era. These terms collectively form a lexicon of endurance that emphasizes bouncing back, withstanding pressure, and fitting in.

“Psychological Toughness” (*xinli renxing*) is the most direct translation of “resilience.” It is presented as a quality to be actively developed. Multiple articles offer advice on how to enhance psychological resilience through self-help techniques (ex. SMHC 2022.06.13), framing resilience as a skillset that can be trained, like a muscle, to become tougher against adversity. Another term often used is *kangnili*, or “resistance force”. This term implies an active capacity to resist and overcome adverse circumstances: “Resilience refers to an individual’s ability to rebound and recover under pressure from difficulties, trauma, or major unexpected events. It is a trait that helps individuals overcome adversity and develop positively” (FGR 2022.03.11). In the Zero-COVID context, however, the target of overcoming subtly shifts. What is to be managed is not the policy-generated condition itself but one’s reaction to it. Finally, there is *xinli tanxing* or “psychological elasticity.” This metaphor suggests the ability to be stretched by stress without breaking and to snap back to one’s original shape. It emphasizes flexibility and recovery.

While the word *shiyingxing* is used, adaptability is more often conveyed through a set of prescribed behaviors and cognitive frames that emphasize acceptance and adjustment to a new, state-defined normal. Acceptance (*jiena*) is the cornerstone of the prescribed adaptive response. Citizens are repeatedly urged to accept their emotions, the situation, and the new rules: “Accept your emotions, and don’t blame yourself” (FGR 2022.05.07); “Accept the circumstances of quarantine, understand your own reactions, and seek positive meaning within adversity” (FGR 2020.01.27). The latter quotation derives from the *Guiding Principles for Emergency Psychological Crisis Intervention during the COVID-19 Epidemic*, issued by the National Health Commission of the People’s Republic of China in the early phase of the pandemic and subsequently reposted by the SMHC. In this framing, adaptation becomes less a question of transforming external conditions than of cultivating an appropriate psychological orientation toward them, positioning acceptance as a central technique of emotional self-regulation under crisis governance. This emphasis effectively recasts state-imposed restrictions as an internal psychological task: “Accept things as they are, do what you ought to do” (*shun qi ziran, wei suo dang wei*). The principle, borrowed from Morita therapy—a Japanese psychotherapeutic approach that emphasizes accepting emotions without attempting to control them (Sugg et al., 2020)—is repeatedly recommended as a practical orientation toward life under lockdowns:

Try applying the core principles of Morita therapy: accept these emotions, learn to live with the “symptoms” (*zhengzhuang*), accept things as they are, and do what you ought to do. Remember, no matter how difficult the present may be, life goes on. Accept the situation, face it directly, and adjust to the changes brought by the pandemic (SMHC 2022.03.22).

Rather than encouraging reflection on the causes of restrictions, these prescriptions directed attention toward maintaining routines and fulfilling obligations within a constrained environment. Adaptation thus functioned as a form of self-governance, aligning personal emotional work with the broader imperatives of crisis management.

### Resilience and adaptability as psychosocial governance

SMHC’s dissemination of these concepts functions as psychosocial governance. Its messaging does not deny distress, but it consistently reframes distress as a matter of emotional regulation rather than a response to policy-produced disruption. The narrative places the burden of psychological survival largely on the individual. The system and its policies are presented as fixed conditions to which the individual must adapt. Personal distress becomes the problem to be managed, rather than the restrictions that generate it.

This encourages a strong focus on internal cognitive and affective control while limiting the political legibility of frustration directed toward authorities. “Whether your life is happy or painful, the disease will have some impact, but it will not affect your whole life. We may not have the final say in what kind of life we want, but there is still a part that we can control” (FGR 2023.01.10). The discourse creates a binary. The “healthy” citizen adapts through routines, and acceptance. “Negative” emotions such as anger, frustration, or despair toward policy are framed as natural but ultimately maladaptive. Rather than openly suppressing dissent, the discourse psychologizes it. A good citizen becomes one who successfully regulates emotional responses to external constraint and expresses gratitude (*gan’en*), echoing the Zero-COVID state’s mobilization of *gan’en* as an affective instrument that casts citizens as emotionally indebted to the state (Sorace, 2020).

SMHC messaging also encouraged citizens to modulate emotional expression through humor and self-deprecation. One post advised readers to “try healthy ways to have less ‘Emo’ and more ‘Hahaha,’ maintaining a positive mindset and a healthy life,” describing an “Emoha” state—a playful mixture of melancholy and laughter. Such expression, “is also a sign of psychological maturity and relatively good social functioning” (FGR 2022.11.18). Drawing on Chinese internet vernacular, “emo” refers to a state of sadness, emotional vulnerability, or moodiness, often expressed in a self-aware and mildly ironic register rather than as a sign of severe psychological distress. This framing normalized mild distress while redirecting it into socially acceptable forms of ironic self-awareness, turning humor into another technique of emotional regulation.

The theoretical framework of the SMHC’s discourse is best understood through its practical, repeated prescriptions. Confinement in the form of prolonged lockdowns and quarantine measures is re-signified as an opportunity for self-development rather than a coercive restriction. The burden of action is located at the level of self-regulation even though the disruption itself is policy-generated: “If you encounter closed-loop management (*bihuan guanli*), you can also improve your mood by adjusting your mindset and perspective on things—’a loss may turn out to be a gain’!” (FGR 2022.03.16). Adaptation thus becomes a matter of recalibrating expectations and emotions so that disruptive conditions can be lived with rather than contested.

One practical technique repeatedly recommended to support this emotional recalibration is the cultivation of everyday “rituals” designed to stabilize mood and restore a sense of order within confinement:

Rituals are like cardiotonic and heart-stabilizing agents (*qiang xin wen xin ji*) that can soothe emotions. Rituals were most often used in scenarios where humans resisted natural disasters and supernatural phenomena, helping them overcome the “sense of helplessness” in understanding the world, which has evolutionary significance. Current scientific discoveries show that rituals can activate the dopamine reward system in the individual’s brain, thereby soothing anxiety and unease and stimulating pleasant emotions…. Rituals are like a feast of positive emotions, promoting interpersonal harmony (FGR 2021.02.14).

Here rituals are framed as therapeutic technologies for managing uncertainty. By invoking neurobiological mechanisms such as dopamine activation, the text recasts ordinary routines as evidence-based techniques for emotional stabilization. The invocation of interpersonal “harmony” (*hexie*) is politically significant, as the construction of a “harmonious society” (*hexie shehui*) has long functioned as a central state slogan in contemporary China (Han, 2008). Ritualized self-care thus aligns personal well-being with the broader political imperative of maintaining social order and stability

Several articles promoted a “psychological stress vaccine” (*xinli yali yimiao*), a form of stress inoculation training designed to help citizens cope with quarantine, infection risk, and uncertainty. Borrowing metaphors from behavioral immunology, the program framed resilience as a kind of psychological immunity that could be cultivated through recognizing stress reactions, learning coping techniques, and repeatedly practicing emotional self-regulation (FGR 2022.04.22). Pandemic disruptions were thus addressed not through structural change but through the cultivation of psychological preparedness and adaptive self-management.

Such advice exemplifies what Foucault (1988) calls “technologies of the self”: practices that encourage individuals to regulate their emotions, maintain routines, and cultivate a sense of control within conditions they cannot change. Resilience thus appears less as collective adaptation or structural transformation than as a form of privatized self-governance, outsourcing stability to individuals who internalize discipline and reinterpret constraint as psychological strength.

Perhaps the most explicit fusion of psychological and political discourse appears in a post summarizing a speech by SMHC Secretary Xie Bin, the same leader who called for “limiting your soul’s desire for freedom” during the Shanghai lockdown: “Have hope (*pantou*), let go (*fangxia*), be self-disciplined (*zilü*), have a vision (*nianxiang*)” (SMHC 2022.03.22). This four-point framework encapsulates the logic of governed adaptability. Citizens are encouraged to accept reality, maintain self-discipline, and find meaning within existing constraints, transforming uncertainty into a manageable psychological condition. Just as a vaccine protects the body from a virus, acceptance of state policy is framed as the necessary injection to protect the mind from dissent and the social body from disorder. Mental health is no longer just aligned with policy compliance; it is *defined* by it.

## Quiet Negotiations: Psychological Counsellors and the Fragmented Landscape of Resilience in Zero-COVID Shanghai

Interviews with psychological counsellors reveal a far more complex and often contradictory landscape of resilience and adaptability in Zero-COVID Shanghai. While there is some overlap with the SMHC’s emphasis on endurance and adjustment, counsellors’ accounts complicate these notions by highlighting questions of agency, trauma, community, and the contested meaning of mental health in crisis.

Some counsellors echoed official narratives of endurance, but even when they did so, their accounts often carried different emphases. Yumei (38), for instance, spoke of an ingrained Chinese capacity to endure hardship: “Chinese people have a stronger adaptability… in our bones lies the ancestral spirit of enduring hardship… We don’t want to suffer, but if we must, we will adapt” (Int 16). Wanshu (40) similarly observed that “Chinese are used to living in environments marked by constraints, so they are quite good at adapting” (Int 15). These remarks resonate with state narratives of endurance but also suggest a historically sedimented habituation to constraint.

At the same time, counsellors’ professional training shaped how they operationalized these categories. Psychoanalytically oriented practitioners foregrounded reflective processing of trauma; cognitive behavioral (CBT) practitioners emphasized skills and routines; humanistic therapists emphasized relational engagement; sports psychologists stressed performance and endurance. These differences matter because they show that “resilience” was not a uniform professional object, but a term refracted through multiple therapeutic epistemologies.

Despite describing lockdown life as “emptiness” (*wuxu*), 46-year-old Liu emphasized the professional necessity of emotional management: “I knew I had to quickly regulate (*tiaojie*) my emotions, otherwise I couldn’t accompany my clients… so from the beginning I contacted an organization in Beijing and had ten individual counselling sessions.” Her proactive engagement with supervision and peer networks illustrates adaptation as resource-seeking, a practice that extends beyond individual psychological traits. In her metaphorical framing, the pandemic is “lurking silently around society, moistening everything (*runwu xiwusheng*)” (Int 17). Originating in a poem by the Tang dynasty poet Du Fu, *runwu xiwusheng* is an idiom increasingly appropriated in contemporary Party discourse to describe the subtle, gradual diffusion of ideological influence and political messaging through everyday life (Bandurski, 2024). By invoking this expression, Liu portrays COVID not simply as an epidemiological threat but as a pervasive force that quietly permeates society, highlighting the diffuse and cumulative nature of psychological stress under prolonged pandemic governance—dimensions largely absent from official public narratives.

Several counsellors pointed to the psychic toll of opacity and uncertainty. As one counsellor put it, “The pandemic had a traumatic effect on people… especially the scarcity of information shared by the government” (Int 16). Others stressed how the experience lingered well beyond the lifting of restrictions, leaving residents “like birds startled by a bow (*jing gong zhi niao*)… panicking at the slightest stir.” For this counsellor, the city itself no longer felt familiar: “Is this still my hometown?... Many things are gone; trauma still surfaces” (Int 17). Wenwen (30), a sports psychologist in her thirties, gave a visceral account of collective breakdown: “During lockdown, many had emotional breakdowns… screaming, crying, running naked, arguing… the atmosphere was very strange.” According to her, the problem was not individual weakness but the stripping away of agency itself: “It was like having your face stepped on. No amount of positivity could change that” (Int 19). Such formulations directly challenge the official premise that appropriate self-adjustment is the primary marker of psychological health. From the counsellors’ perspective, what appeared publicly as “adaptation” could also describe pathological accommodation to escalating harm.

### The Complexity of Agency: Between Adjustment and Helplessness

The SMHC narrative is built on a notion of proactive adjustment. The counsellors, however, painted a picture where agency was often constrained, and adaptation was less a choice than a necessity born of helplessness, as exemplified in the quote opening this article: “I don’t know whether to call it ‘adaptation’ or ‘helpless forced acceptance’” (Int 20). While SMHC framed resilience as a universal capacity, counsellors exposed its uneven distribution. Wang Jie (46) identified two categories of response to pandemic uncertainty: those who accept it and those who were destabilized by “wind moving the grass” (*feng chui cao dong*) (Int 17). Mou (39) noted that the “chronicity” (*manxinghua*) of the lockdown became a “torment” (*zhemo*), highlighting that its psychological effects often surfaced months later through somatization or emotional disorders (Int 22). Kai (42) insisted: “We are not just individuals with individual psychology, but social beings” (Int 21b). His systemic framing is important because it shifts the analytic focus from individual compliance to relational entanglement. Adaptation, in this view, is mediated by family roles, community resources, social position, and time. Psychological resilience is neither evenly distributed nor immediately visible.

Wenwen argued that the pandemic differed from disasters such as earthquakes because it combined elements of both “natural disaster” (*tianzai*) and “man-made disaster” (*renhuo*). According to Wenwen, while the virus itself was uncontrollable, much of the public’s anger and powerlessness stemmed from policy failures. She noted that people could more readily accept earthquakes because they are “completely beyond human control,” whereas during the pandemic many felt despair in response to officials “using the pandemic to seek personal gain” (*yi yiqing mou sili*) and other perceived policy shortcomings. Her account critiques the state’s response as a form of structural violence, depriving people of “agency” (*nengdongxing)* to meaningfully respond to the restrictions imposed on their everyday lives, making adaptation forced rather than negotiated (Int 19).

Hua Mei (69), a counsellor at a state organization for protection of women, who was originally trained in propaganda work, added yet another dimension. Her background in both Party education and humanistic therapy made her uniquely positioned to bridge state and therapeutic discourses. For her, resilience meant helping clients “organize, refine, and advance” (*zhengli*, *guina*, *tuijin*) their thoughts through attentive listening and empathy. She drew explicitly on humanistic traditions, framing China as “a country of etiquette” (*liyi zhi bang*), where counselling required patient listening as counterpoint to hurried, utilitarian social rhythms. Hua Mei interpreted widespread compliance to harsh anti-pandemic policies as culturally embedded “resignation to adversity” (*ni lai shun shou*) but warned that the “three years of bitterness” (*san nian zhi ku*) of the pandemic had eroded popular trust in government (Int 14).

Jiang Hong (43), a former IT project manager trained in psychoanalysis and analytical psychology, argued that resilience discourse concealed trauma and compliance, masking the erosion of civic rights during Zero-COVID: “This is not adaptation, this is numbing… like boiling a frog in warm water (*wenshui zhu qingwa*), it ultimately becomes alienation (*yihua*).” She directly linked acceptance to authoritarian control: “If people truly got used to the so-called ‘new normal’ (*xin changtai*), it would be a tragedy.” For her, adaptation meant alienation rather than resilience, a lesson she connected to intergenerational transmission (*daijixing de chuandi*) of trauma of the Cultural Revolution and called for recognition “aking to the Holocaust”: Yet she admitted therapeutic challenges: sometimes indulging clients’ anger against the government, even praising their critique, something she acknowledged blurred professional boundaries (Int 20). Her account shows that counsellors did not merely resist official discourse from outside it; they often negotiated it from within therapeutic relationships that were themselves ethically unstable and politically charged.

Counsellors’ professional practices were also reshaped by the pandemic, often complicating efforts to frame their work as fostering “adaptation.” One counsellor noted that many child clients discontinued therapy during lockdown, leaving her working mainly with adults instead (Int 16). For others, the main challenge was economic insecurity. A counsellor who worked with athletes noted that repeated competition cancellations had wiped out two major contracts, leaving him still trying to recalibrate his professional footing (Int 22). Rather than a stable psychological resource, resilience emerged as a precarious process of improvising amid professional and emotional uncertainty.

Reflecting on the post-pandemic period, Wenwen, whom I re-interviewed in May 2023, argued that many people’s desire to “move on” should not be mistaken for recovery. Rather, she understood it as a form of dissociation (*jieli*) that helped people cope with lingering trauma. As she explained, “People’s desire to ‘move on’ in post-pandemic times is a strategy to deal with trauma, it is a way to dissociate. But even if we choose not to think about it, it does not mean that its effects will not linger.” For Wenwen, dissociation was a common psychological mechanism that enabled people to function despite difficult emotions. The problem arose when it became a “normal state” (*changtai*, same as in *xin changtai*, or the “new normal”). Shanghai lockdown as a chronotope: The biopolitics of, denoting a condition that has been rendered ordinary and taken for granted), preventing individuals from recognizing or processing the emotional consequences of their experiences (Int 18b). In her counselling practice, she therefore avoided pushing clients to confront trauma before they were ready. If someone insisted that an event had no impact on them, she preferred to “put it on the side for now,” allowing difficult experiences to be approached gradually and on their own terms. Wenwen’s account suggests that the apparent desire to leave the pandemic behind may represent not recovery but a temporary suspension of suffering. What appears as adaptation or resilience may coexist with unresolved trauma that continues to shape everyday life beneath the surface.

Kai, whom I also re-interviewed in spring 2023, offered a metaphor that captured many counsellors’ reservations about official narratives of resilience. He compared Zero-COVID policies to “wrapping a child completely like an infant in swaddling clothes,” shielding them from external threats, while reopening was like “suddenly pushing a well-protected child out the front door” (Int 21b). People were expected to adapt rapidly to radically altered circumstances without adequate preparation, leaving many disoriented and unsure how to respond. The only option was often to improvise, drawing on whatever coping strategies were available. Kai’s account highlights a recurring theme across the interviews: resilience was less a stable psychological resource than a fragile and uneven process of navigating uncertainty under conditions not of one’s own choosing.

### Negotiating Professional Practice Within and Against State Discourse

Counsellors actively mediated between state discourse and client needs. Wenshan (41) is a counsellor employed in the Public Complaints and Proposals Administration Office, an official body managing the administrative system known as *xinfang*, or “petitioning,” which enables citizens to submit grievances to the government concerning matters like land expropriation, corruption, labor conflicts, or official misconduct. Clients frequently arrived with concrete demands, he noted, but his role was largely to defuse their distress, especially since many of those demands lay beyond what the office could address (Int 23). His position illustrates therapeutic governance in a particularly clear form: counselling becomes part of a bureaucratic apparatus that manages affect when substantive redress is limited. Yet even here, mediation is not mechanical. Wenshan described offering behavioral experiments, structured journaling, and small goal setting—techniques that partially align with official discourse while retaining a degree of professional autonomy.

Yu (39), an accomplished sport psychologist shared that in her psychology classes with athletes she stressed that while “the state says to maintain psychological resilience, … I tell people… resilience doesn’t mean enduring injustice; it means finding methods and resources.” (Int 22). This is a subtle but meaningful shift. Rather than rejecting resilience outright, she redefines it away from stoic compliance and toward practical resourcefulness.

Several counsellors also pushed back against the state’s invocation of “positive energy” (*zheng nengliang*), a discourse promoting optimism, harmony, and emotional self-regulation (Hird, 2018), as the dominant framework for resilience during the pandemic. Jiang Hong argued that “positive energy is sometimes coercive… forcing people to suppress authentic feelings” (Int 20). She critiqued such “manufactured positivity” (Yang, 2022, p. 151) as both a personal defense mechanism and authoritarian strategy and recalled a client who listened to *zheng nengliang* songs daily, masking pain rather than confronting it. For her, therapy’s purpose was to uncover, not conceal, the negative. This tension between officially prescribed optimism and privately experienced distress can be understood as a form of affective dissonance, in which individuals are encouraged to display emotional alignment with dominant narratives while internally experiencing contradictory feelings (Iskra, 2026). Hua Mei likewise stressed that counselling must make room for difficult feelings rather than simply push clients toward adjustment. “We cannot just teach people to ‘adapt,’” she insisted; instead, practitioners should help clients “face (*miandui)*, express (*biaoda*), and process (*chuli*) emotions” (Int 14). These accounts show that counsellors rarely rejected the vocabulary of resilience outright. More often, they reinterpreted it by insisting that adaptation without recognition of grief, anger, or harm was psychologically thin and ethically suspect.

### Resilience Beyond the Individual: Community, Gender, and Systemic Constraints

The counsellors regularly pointed to relational and gendered dimensions of resilience that the official narrative downplays. Most counsellors highlighted that under the Zero-COVID regime community ties strengthened. Wenshan observed that “people could seek more help within their residential compound, something they hadn’t realized before” (Int 23). Counsellors also highlighted the important role of young volunteers in fostering collective resilience as they took care of neighbors diagnosed with COVID.

Many argued that confinement restructured family roles, making men’s domestic presence a source of new conflicts and women’s emotional outbursts. Hua Mei, a counsellor at the state organization protecting women, reported that domestic violence cases surged, interpreting women’s emotional outbursts as “triggers” (*chudian*) for male violence, while simultaneously advising them to seek police protection (Int 14). Hua Mei’s account thus reveals a paradox within the discourse of resilience: while acknowledging the surge in domestic violence and advising women to seek institutional protection, she simultaneously framed women’s emotional reactions as triggers of male aggression, subtly recasting women’s endurance and emotional restraint as necessary components of coping with violence.

Jiang Hong offered vivid stories from the lockdown period: a 70-year-old woman demanding an exit pass for exercise, whose defiance towards security guards secured permits for all residence, as well as her own act of secretly taking her adolescent daughter outside the residential compound, despite restrictions, countering her desperate cry of “This is driving me to death” (*bisi wo*) (Int 20). These accounts frame resilience not simply as quiet endurance but as relational action, small-scale defiance, and care in conditions of constraint.

Gendered burdens were central in counsellors’ accounts. They highlighted how women bore disproportionate childcare and household responsibilities during lockdown, like Mou who argued that women’s resilience often went unrecognized: “Many women were providing invisible psychological support… their adaptation is unseen, but they were the most exhausted.” (Int 22). Wang Jie emphasized the gendered dimension of grassroots mobilization in urban neighborhoods. She pointed to *tuanzhang*, or grassroots neighborhood coordinators, often self-appointed or community-elected, who organized group purchases, distributed supplies, and mediated between residents and local authorities under lockdown. Wang Jie highlighted that a significant majority of *tuanzhang* were women, including many housewives, which in her view showcased their adaptability (*yingbian nengli*, lit. “ability to respond to changes”) and energetic initiative in the face of food shortages (Int 17).

Taken together, these accounts reveal a form of bottom-up, relational resilience operating alongside—and often despite—official messaging. Resilience here was socially mediated and unevenly distributed, dependent on family position, gendered labor, neighborhood resources, and the capacity to improvise. Such accounts complicate both the official universalism of psychological toughness and narrower models of resilience as individual coping.

## Conclusion: Resilience Multiple. Therapeutic Governance, Counsellors’ Practice, and Global Contestations

This article argued that during Shanghai’s Zero-COVID period, resilience and adaptability functioned not as stable psychological traits but as contested idioms through which mental health, citizenship, and crisis survival were negotiated. State-affiliated messaging from the Shanghai Mental Health Centre promoted psychological toughness, and flexibility, as well as acceptance, gratitude, and disciplined self-adjustment as proper responses to lockdown. Counsellors, by contrast, often used the same vocabulary while quietly reworking it through clinical idioms of trauma, numbness, delayed collapse, relational care, and professional ambivalence.

The point is not that official discourse was wholly hegemonic or that counsellors simply resisted it. Rather, resilience circulated through a wider field of actors—institutions, platforms, practitioners, clients, and communities—who gave it different meanings. While SMHC materials framed distress as a matter of emotional self-management and linked well-being to responsible cooperation, counsellors encountered the limits of this framing in practice. Their accounts foregrounded uncertainty, structural harm, family conflict, gendered exhaustion, and forms of adaptation that often resembled reluctant endurance rather than confident coping.

Seen in this light, counsellors emerge as mediating experts whose work illuminates the contradictions of therapeutic governance. Increasingly drawn into state-linked crisis response through outsourcing and community service, they nevertheless confronted the gap between policy-prescribed adjustment and lived distress. Some reproduced official language, others redefined resilience as resourcefulness rather than submission, and still others viewed adaptation itself as troubling when it signified numbing, alienation, or the normalization of rights erosion.

The Shanghai case also contributes to resilience scholarship by showing that resilience becomes politically meaningful through its translation into specific institutional and moral worlds. Rather than contrasting “Chinese” and “Euro-American” resilience, the analysis highlights how psychological categories are mobilized, by whom, and toward what ends. In Shanghai, resilience served projects of psychosocial governance while also being re-signified through therapeutic practice and everyday survival.

Finally, the ethnography shows that resilience under Zero-COVID was embodied, relational, and unevenly distributed. It appeared in the invisible emotional labor of women, in neighborhood mutual aid, in professional self-regulation, and in the afterlives of lockdown trauma—sometimes sustaining survival, sometimes masking slow forms of collapse. Counselling emerged as a site of quiet negotiation between these competing meanings: while the state promoted resilience as disciplined adaptation to crisis, counsellors appropriated and reinterpreted this vocabulary in their efforts to address human suffering. Resilience thus appears not as a singular virtue but as a “resilience multiple” (Zebrowski, 2025): a politically charged language through which care, compliance, critique, and endurance were continually renegotiated.

## Data Availability

Due to the data’s political sensitivity and in order to protect research participants, the research data generated in this study will not be made publicly available.
